# Human Immunodeficiency Virus (HIV)-Infected Patients Accept Finger Stick Blood Collection for Point-Of-Care CD4 Testing

**DOI:** 10.1371/journal.pone.0161891

**Published:** 2016-08-24

**Authors:** Géraldine Daneau, Natasha Gous, Lesley Scott, Joachim Potgieter, Luc Kestens, Wendy Stevens

**Affiliations:** 1 Immunology Unit, Department of Biomedical Sciences, Institute of Tropical Medicine, Antwerp, Belgium; 2 Department of Molecular Medicine and Haematology, School of Pathology, Faculty of Health Sciences, University of Witwatersrand, Johannesburg, South Africa; 3 Department of Haematology, National Health Laboratory Services – Tshwane Academic Division and University of Pretoria, Pretoria, South Africa; 4 Department of Biomedical Sciences, University of Antwerp, Antwerp, Belgium; 5 National Priority Program of the National Health Laboratory Service, Johannesburg, South Africa; Rush University, UNITED STATES

## Abstract

**Introduction:**

HIV-infected patients require antiretroviral treatment for life. To improve access to care, CD4 enumeration and viral load tests have been redesigned to be used as point-of-care techniques using finger-stick blood. Accurate CD4 counting in capillary blood requires a free flowing blood drop that is achieved by blade incision. The aim of this study was to assess the attitude of the patients toward blade-based finger-stick blood donation.

**Methods:**

Four hundred and ninety-nine patients were included (299 patients from South Africa and 200 from Belgium). They completed a questionnaire to express their preference for finger stick or venipuncture, after undergoing both. The South African patient cohort was divided in two groups, receiving either single or multiple finger stick for CD4 and other HIV-related tests. The Belgian patients received a single finger stick for CD4 testing, and were asked to respond directly and again after two days.

**Results:**

The majority of the patients preferred the finger stick to the venipuncture. The perceived pain using the blade was superior to a small needle, but similar to a large needle. They preferred up to three finger sticks over one venipuncture. Up to 30% of the patients changed their mind over two days. The main reason for choosing a finger stick was continued bleeding after venipuncture. The most cited objection to finger stick was pain/soreness.

**Conclusion:**

Patient perceptions support the implementation of donating capillary blood with blade-based finger stick during CD4 point-of-care testing.

## Introduction

Human immunodeficiency virus (HIV) infects 35 million people worldwide [1]. Availability and safety of antiretroviral treatment (ART) has increased over the years. However, only 36% of HIV-infected people in low- and middle-income countries are receiving ART, and the World Health Organization (WHO) expects an additional 11 million people eligible for treatment by increasing the threshold of treatment initiation from 350 to 500 CD4 cells/mm^3^ [2]. In the future, all HIV-seropositive patients will be treated as early as diagnosed with HIV, through the “Test and treat” strategy. While this approach will no longer use the CD4 counts for treatment eligibility, the implementation on worldwide scale seems far from now [2]. However, with the scale up of viral load testing, CD4 cell enumeration is already becoming less utilized to monitor ART [3]. Fortunately, the development of Point-of-care (POC) testing for CD4 has expanded services to patients in remote areas, and has been shown to decrease patient pre-treatment loss-to-follow-up as CD4 results are known immediately [4]. POC technologies are designed to be used outside of a central laboratory, and typically do not require cold chain transport of reagents, or a stable electricity or water supply [5]. POC testing is designed to be simple thus allowing health care workers like nurses and physicians with a basic training and no prior laboratory experience to perform testing on minimally invasive specimen types such as finger stick/capillary blood [6]. Capillary sampling from the finger is less invasive and requires less skill than obtaining a sample via venipuncture, and the required collected blood volume is smaller. However, collecting capillary blood for CD4 counting is different than for instance for HIV diagnosis, malaria or glucose testing. The CD4 count in a blood drop should be equivalent to the CD4 count in venous blood, and therefore requires a larger stick, creating a slice using a 1.5 mm blade instead of a needle, to allow blood to flow more freely [7]. Moreover, collection and wound handling should be done with care to limit exposure of the healthcare worker to infectious blood.

Recommendations for POC testing for large-scale implementation programs are based on practical aspects, but mostly overlook patient acceptance, mainly/especially when multiple tests are required. Indeed, the ART treatment and monitoring guidelines, including the 2015 South African treatment guidelines, propose multiple assays for ART initiation, including monitoring drug toxicity [3;8].

Patient preferences comparing finger stick with venipuncture have been investigated for repeated use applications, like determining blood immunosuppressant concentration in transplanted patients, or International Normalized Ratio (INR) in anticoagulant therapy [9–12]. In all these studies, patients preferred finger stick over venipuncture. Diabetic patients perceived the discomfort of a finger-stick as similar to venipuncture, both with a high satisfaction rate [13]. Patient’s preference for finger stick or venipuncture has never been investigated in the case of CD4 testing or similar blade incision.

In this study, we investigate whether patients presenting for a CD4 test and other HIV related tests would prefer venipuncture or a finger stick, or even multiple finger sticks in the case of multiple POC testing.

## Material and Methods

During the evaluations of a POC CD4 instrument (not shown here), we added a questionnaire to be completed by the patients to obtain feed-back on their finger stick experience. Patients were enrolled at two study sites: the Comprehensive Care Management and Treatment clinic (CCMT) at Tshwane District Hospital in Pretoria, South Africa, and the HIV clinic at the Institute of Tropical Medicine (ITM) in Antwerp, Belgium, when visiting for ART initiation/monitoring, with a requested CD4 enumeration. Questionnaires in all applied languages are available as Supportive Information.

In Tshwane (Pretoria), the study was divided in two groups. One group of 150 patients had multiple POC testing performed by a single finger stick using a selected lancet due to the amount of blood it produces from the blade (Pima CD4 testing lancet), namely the Sarstedt Safety lancet (Sarstedt Group) (Group 1). A second finger stick was only performed if insufficient blood was available to complete all the POC tests requested. The second group of 150 patients had multiple POC testing performed, each POC test on a separate finger stick (needle and blade based lancets) based on respective standard operating procedures with manufacturer recommended lancets (Group 2) (see Table 1). The doctor requested the test repertoire as per national treatment guidelines at the time of the study, and trained study staff collected finger stick specimens, and performed POC testing in a designated POC testing room. A venipuncture specimen was also sent to the laboratory as per standard of care and for clinical decision making. After the finger stick and venipuncture, the nurse completed a short questionnaire to evaluate the patient’s experience of finger stick blood collection. Feedback from the two POC study nurses was also obtained post-study to gain insight on their experience of performing multiple versus single finger sticks for POC testing on patients.

**Table 1:** Lancets used for multiple POC during study in Tshwane, as recommended by the respective manufacturers of the POC test.

Testing group	Lancet used	Lancet Specifications	Parameter
Group 1 and 2	Sarstedt safety lancet	1.6mm depth x 1.5mm **blade**	**CD4 count**
Group 2	HemoCue safety lancet	2.25mm depth x 23G **(large) needle**	**Haemoglobin**
Group 2	Roche Accu-Chek Softclix Pro lancet	1.7mm depth x 28G **(small) needle**	**ALT and creatinine**

ALT = Alanine aminotransferase

In Antwerp, 200 patients were enrolled. Besides routine venipuncture, finger stick was performed with the BD Microtainer contact-activated lancet 2.0 mm depth x 1.5 mm blade (Becton Dickinson, Plymouth, UK). Patients were immediately asked to complete the questionnaire (available in Dutch, French, and English) post-finger stick. In addition, they were given a second questionnaire to be completed two days after the finger stick, or later when all symptoms would have disappeared, and either return the questionnaire during their next visit to the clinic or send it back by recipient-paid post; a study code linked the two questionnaires from each patient. On both questionnaires, they could thick one or more reasons for their choice, and add comments.

Both Tshwane and Antwerp study nurses performing the finger stick shared their experience with a verbal feed-back session.

Statistics were performed with MedCalc Version 10.0.2.0, applying Chi-square test to compare the different answers between groups. Percentages are shown with two significant digits.

Studies were respectively approved by the University of the Witwatersrand Human Research Ethics Committee (protocol number M120143), and by the Institutional Review Board of ITM and the Ethical Committee at University of Antwerp Hospital (protocol number B300201420409), and all patients signed an informed consent form.

## Results

### Patient experience of multiple finger sticks

Of the 299 patients who answered the questionnaire in Tshwane, 149 had a single finger stick (by blade) for multiple POC tests (group 1), and 150 had multiple finger sticks (needle and blade-based lancets; group 2). Patients in the second group had up to 6 finger sticks performed, as reported previously [14].

**Fig 1:**
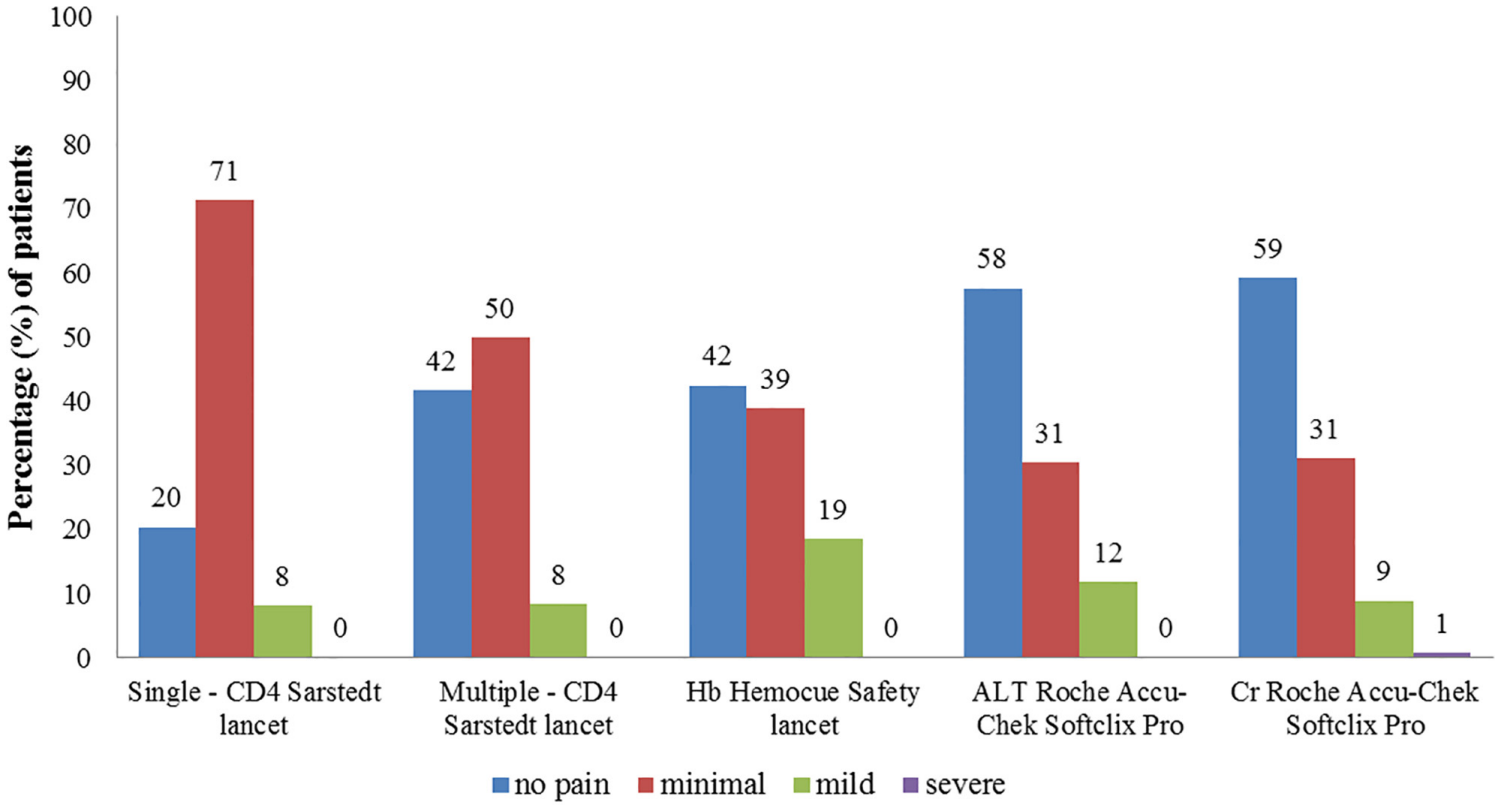
Pain levels experienced by the patients in the single and multiple finger stick groups, for the different lancets used. CD4 = CD4 T cell enumeration (with blade); Hb = haemoglobin (with large needle); ALT = Alanine aminotransferase (with small needle); Cr = creatinine (with small needle); n = 149 patients for single and 150 for multiple finger stick groups.

A summary of the patient responses to the questions asked by the study nurse are shown in Table 2. The perceptions after the finger stick were similar between the first and the second group, and with an equal proportion of patients experiencing pain in the finger following finger stick. In both groups, a minority of patients felt that finger stick was worse than venipuncture (p = 0.002 in group one; p < 0.0001 in group two). In the second group, the pain was reported differently for the different lancets used (p = 0.0064) (Fig 1). Specifically, when comparing lancets, the CD4 Sarstedt lancet (blade), which performs a finger slice, was reported as being more painful (p = 0.0063) than the Roche Accu-Chek Softclix Pro system (small needle), and similar to the Hemocue Safety lancet (large needle) (p = 0.055). Interestingly, the pain reported after receiving finger stick by blade (for CD4) was lower in the multiple finger stick group (group 2) than in the single finger stick group (group 1) (p = 0.0008). Finally, patients who had experienced multiple finger sticks would prefer to have up to three finger sticks instead of a venipuncture performed for blood collection, while patients receiving only one finger stick would limit the advantage to one or two sticks (p < 0.0001).

**Table 2:** Responses to questionnaire in Tshwane from patients who gave a venipuncture blood sample after a single (group 1) or after multiple (group 2) finger sticks.

Question asked	Responses	First group: Single Finger stick [n (%)]	Second group: Multiple Finger sticks [n (%)]			
**How does your finger feel after the finger stick?** *	Fine	71 (54%)	**80 (53%)**			
	Sore	59 (45%)	**70 (47%)**			
	Don’t know	1 (0.8%)	**0 (0%)**			
**Was having a finger stick worse than having blood taken from your arm?**	Yes	34 (29%)	**35 (23%)**			
	No	70 (47%)	**78 (52%)**			
	Felt the same	43 (29%)	**36 (24%)**			
	Don’t know	2 (1.3%)	**1 (0.7%)**			
**Please rate the pain level from the finger sticks you received**. †		**Single lancet (CD4)**	**CD4**	**Hb**	**ALT**	**Cr**
	1 = no pain	30 (20%)	45 (42%)	48 (43%)	68 (58%)	**80 (59%)**
	2 = minimal	105 (71%)	54 (50%)	44 (39%)	36 (31%)	**42 (31%)**
	3 = mild	12 (8.2%)	9 (8.3%)	21 (19%)	14 (12%)	**12 (8.9%)**
	4 = severe	0 (0%)	0 (0%)	0 (0%)	0 (0%)	**1 (0.7%)**
**Which statement is the most correct to you?** ‡	I would prefer having x1 finger stick before having blood taken from my arm	97 (66%)	**16 (11%)**			
	I would prefer having x2 finger sticks before having blood taken from my arm	51 (35%)	**43 (31%)**			
	I would prefer having x3 finger sticks before having blood taken from my arm	0 (0%)	**76 (54%)**			
	I prefer blood taken from my arm	0 (0%)	**6 (4.3%)**			

CD4 = CD4 T cell enumeration (with blade); Hb = haemoglobin (with large needle); ALT = Alanine aminotransferase (with small needle); n = 149 patients for first group and 150 for second group; some questions were not answered by participants, reducing number of answers:

* n = 131 for first group;

^**†**^ n = 147 for first group, and 108 for CD4, 113 for Hb, 118 for ALT, and 135 for Cr for second group;

^**‡**^ n = 148 for first group and 141 for second group.

### Patient perception of finger stick over time

Of 200 patients in Antwerp who completed the questionnaire immediately after blood collection (single stick for CD4), 119 (60%) completed a second questionnaire after two or more days following the finger stick to re-assess their preference. The median age of the participants was 46 years (range 19–83 years), and 70.5% were male. The large majority completed the first questionnaire in Dutch (74.5%), followed by French (15.5%) and then English (10.0%). A similar distribution was obtained for patients who completed both questionnaires (82.4% Dutch, 8.4% French, 9.2% English, p > 0.05). More patients preferred a finger stick over venipuncture (77 [38.5%] vs 51 [25.5%], p = 0.0074) whereas a similar proportion of patients (71 [36.5%]) had no preference.

**Fig 2:**
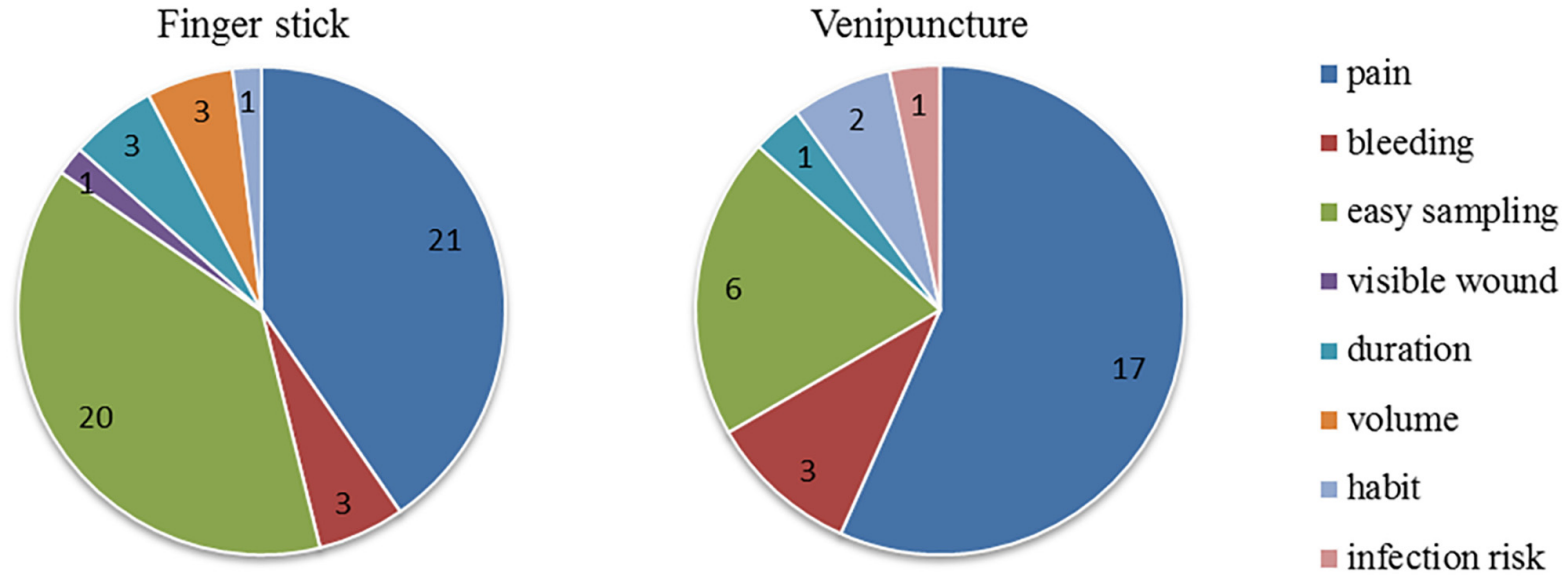
Reasons provided by 69 patients for their preference. Preference for either finger stick (left, n = 41) or venipuncture (right, n = 28) blood collection, immediately post-finger stick. Numbers are absolute number of patients mentioning that reason.

Only 54% (69/128) of patients who indicated a preference for either finger stick or venipuncture provided reasons for their preference (Fig 2). The main reason to prefer either a finger stick or venipuncture was perception of pain. In addition, finger stick was reported by patients as being an easier method to obtain a blood sample, with some individual comments explaining that finger prick was “quick” or “simple”, or, on the contrary, that their veins are difficult to find.

Interestingly, 30% (36/119) of the patients changed their mind after two days, with similar switches from finger to venous and vice versa. For patients who switched their preference from finger stick to venipuncture, pain was given as the major reason. The reason for patients switching preference from venipuncture to finger stick was continued bleeding after venipuncture. Continued bleeding after finger stick was also the major reason to acquire a preference for venipuncture for patients who first had no preference for either. All reported reasons are summarized in Table 3.

**Table 3:** Reasons mentioned by the 36 patients in Antwerp who changed their preference after 2 days.

Change from	To	n changes	n with reason(s)	Pain	Bleeding	Easy sampling	Visible wound/ annoying tape
**Finger stick**	**Venipuncture**	8	8	7	4		3
	**No preference**	8	5	3	1		3
**Venipuncture**	**Finger stick**	3	2		1	1	
	**No preference**	4	1		1		
**No preference**	**Finger stick**	6	2	1			1
	**Venipuncture**	7	6	2	5		1

Globally, the pain after finger stick lasted more than 2 hours for 12 out of 28 patients mentioning the duration, while only one out of 16 patients reported pain 2 hours after venipuncture (p = 0.027). In addition, wound bleeding lasted longer after a finger stick than after venipuncture: 16 out of 28 reported continued bleeding 5 minutes or even longer after the finger stick whereas 2 patients out of 13 reported continued bleeding 5 minutes after venipuncture (p = 0.03).

### Nurses experience of performing a finger stick

The three Antwerp study nurses had a preference for venipuncture blood collection as they perceived they were more exposed to infectious blood from patients by taking a blood sample by finger stick than by venipuncture. Indeed, finger stick leads to external bleeding, while venipuncture captures blood directly in the tube, and only syringe removal is considered a risk exposure. While the wound is on the hands rather than in the elbow pit, nurses were also concerned about peer contamination in case of wound reopening and bleeding, mainly for patients who work with their hands. In addition, they felt it took longer to take a finger stick blood specimen than venous blood as it was not yet a routine well-practiced procedure in this environment (no exact duration measured).

The two South African study nurses indicated that the single and multiple finger stick arms were practical to perform in terms of collecting blood and performing the POC testing in between, in comparison to venipuncture. However, both nurses experienced that single finger sticks were noticeably easier and less time consuming, as multiple finger sticks took longer to perform, led to more bleeding requiring more gauze and plasters, and were visibly more uncomfortable for the patients. The risk of exposure to infected blood of the healthcare worker and the risk of specimen cross contamination was also higher with multiple finger sticks as the nurses had to change gloves and clean the instrument and work surfaces more frequently during the day. The South African study nurses indicated that patients were very willing to participate regardless of whether they would receive single or multiple finger sticks, as their health and obtaining immediate results were of primary importance.

## Discussion

South Africa has the largest ART treatment program in the world with almost 3 million people with HIV receiving treatment [15]. Those numbers are expected to increase as this represented a coverage of less than 50% of the HIV-infected patients eligible for ART according to the 2013 WHO guidelines [1;3;16]. ART initiation involves a combination of assays and would require a multi-disciplinary approach if POC instruments were introduced. Our study results are in support of the ongoing process of implementing finger stick, as the majority of patients would prefer a finger stick over venipuncture to give a blood specimen. If POC CD4 counting is combined with other finger stick tests, still more than 50% of the patients in Tshwane preferred multiple finger sticks over venipuncture. As finger stick is already performed during HIV counselling and testing (HCT), those patients were familiar with the approach of capillary blood sampling. Patients in Antwerp were also more in favour of a finger stick than of venipuncture, despite the fact that pain and bleeding were worse after a finger stick than after venipuncture. Moreover, the majority of patients stuck to their preference after a few days, so their first impressions were not modified by any long-lasting effects. This follow-up study with a high response rate was possible thanks to our stable patient cohort, and the ongoing advocacy to promote the involvement of patients in research. The patient population in the Antwerp clinic is diverse, with around half of the patients (56%) born with Belgian nationality, a quarter from Sub-Saharan Africa (26%), and some from Asia (4%) (cohort data 2013). These proportions might vary in our study, as 75% of the participants answered the questionnaire in Dutch, the official regional language, meaning they have lived in the region for at least a number of years, while immigrants usually speak French or English with the nurses. This diversity broadens our results to different culture perspectives.

Frequent and life-long monitoring through blood tests is not specific to HIV patients. Follow-up of immunosuppressive drug concentration in transplanted patients, INR in case of anti-coagulant therapy, or glucose levels for diabetic patients have already applied finger stick to collect blood, including at home by the patient himself/herself with direct measurement or sending dried blood spots (DBS) to a laboratory [12;17;18]. The use of needle-based finger stick rather than venipuncture was accepted by most patients [9–12]. Nevertheless, we showed that the pain level was not satisfactory for all patients, so we may also need to investigate alternatives, such sticking in the forearm, which previously appeared to be less painful for both adults and children with diabetes [19;20]. However, accuracy of blood collection at a different body site should then be further investigated, and be applicable for blood collection in cartridges. The finger stick at home is hardly applicable for CD4 count, as the whole blood should be measured directly or within the 2 hours with the current POC technologies (Pima CD4, Alere; FACSPresto, BD Biosciences). Nevertheless, it could be developed for HIV patients receiving anti-retroviral treatment to follow their viral load or the drug concentrations by sending dried blood spots to a laboratory [21].

Patient’s opinions bring an additional element of discussion around finger stick versus venipuncture, strengthening the idea that a finger stick is less invasive. Finger stick can be applied in settings where no phlebotomist is present, while training of staff for taking blood from a finger stick may be easier than for venipuncture. In addition, the capillary blood sample is directly measured, while the venous sample has to be transferred to the disposable cartridge or appropriate testing material (usually done by a technician in a laboratory environment, using a pipette). On the contrary, the test precision and accuracy reported after finger stick for CD4 counting was reported to be slightly inferior in finger stick blood than in blood obtained by venipuncture in some settings [22–24]. Those parameters could be improved by better training of local staff, highlighting that finger stick for quantitative CD4 enumeration is not similar to finger stick blood for a thick smear (malaria) or for blood sugar measurements (diabetes). This was indeed the appreciation of our nurses, who did not feel comfortable with the different approach. Nurses or skilled persons who are going to perform the finger stick for CD4 testing should be properly trained to ensure test accuracy as well as biosafety and possibly decrease pain level.

In conclusion, the majority of patients are in favour of a finger stick for CD4 enumeration, rather than giving a blood sample via venipuncture, including for multiple POC tests. In addition, the diversity of the two population groups, from different countries, did not alter perception. These results support the increased use of capillary blood during POC testing outside the classic laboratory and/or clinic structure to facilitate roll-out in different settings.

## Supporting Information

S1 File Questionnaire for group 1 in South Africa. Patients with single finger stick.
(PDF)

S2 File Questionnaire for group 2 in South Africa. Patients with multiple finger stick.
(PDF)

S3 File First questionnaire in Antwerp (English). For preference immediately after finger stick.
(PDF)

S4 File First questionnaire in Antwerp (Dutch). For preference immediately after finger stick.
(PDF)

S5 File First questionnaire in Antwerp (French). For preference immediately after finger stick.
(PDF)

S6 File Second questionnaire in Antwerp (English). For preference two days after finger stick.
(PDF)

S7 File Second questionnaire in Antwerp (Dutch). For preference two days after finger stick.
(PDF)

S8 File Second questionnaire in Antwerp (French). For preference two days after finger stick.
(PDF)
