# Supplementary material for: Human Immunodeficiency Virus (HIV)-Infected Patients Accept Finger Stick Blood Collection for Point-Of-Care CD4 Testing
Source: PLoS One. 2016 Aug 24;11(8):e0161891. doi: 10.1371/journal.pone.0161891 (PMC4996420; doi:10.1371/journal.pone.0161891)
Supplement: S1 File — Patients with single finger stick. (PDF) [file pone.0161891.s001.pdf]

Appendix 3B: Questionnaire for patients 151-300 recruited on <u>shortcut SOP</u> arm [one finger stick performed for all tests requested]

<u>PATIENT INFORMATION</u>

Patient number:_______________________

Age:_________

Sex: ☐ M ☐ F

<u>PATIENT PARTICIPATION</u>

1. Did the patient consent to participate in the study: ☐ Y ☐ N

2. For both the patients that consented into the study <u>AND</u> those that did not consent, please tick which tests were requested by the doctor for the patient:

- ☐ CD4
- ☐ Hb
- ☐ ALT
- ☐ Creatinine

3. For <u>patients who consent</u> into the study please continue to ask the patient the questions on page 2 section 3 below **after taking their blood** for POC testing

3.1. How did it feel after having your finger stick?

- ☐ It was fine
- ☐ It hurt
- ☐ I don't know

3.2. Was finger stick worse than having your blood taken from your arm?

- ☐ Yes
- ☐ No
- ☐ It felt the same
- ☐ I don't know

3.3. Please rate the level of pain you experienced for each of the finger sticks, on a **scale of 1 to 4**

(where 1 = no pain; 2 = minimal pain; 3 = mild pain; 4 = severe pain)

CD4 scale_____

Hb scale_____

ALT scale_____

Creatinine scale_____

3.4. Which is most correct to you?

- ☐ I prefer x1 finger stick over x1 needle draw
- ☐ I prefer x2 finger sticks over x1 needle draw

4. Was more than one finger stick needed to perform the POC tests requested?

☐ Y ☐ N

NURSE COMMENT SECTION

Do you have any additional comments or things you would like to share?

____

____

____
