# Supplementary material for: Human Immunodeficiency Virus (HIV)-Infected Patients Accept Finger Stick Blood Collection for Point-Of-Care CD4 Testing
Source: PLoS One. 2016 Aug 24;11(8):e0161891. doi: 10.1371/journal.pone.0161891 (PMC4996420; doi:10.1371/journal.pone.0161891)
Supplement: S2 File — Patients with multiple finger stick. (PDF) [file pone.0161891.s002.pdf]

Appendix 3A: Questionnaire for first 150 patients recruited on standard SOP arm [a finger stick per test requested]

PATIENT INFORMATION

Patient number:_______________________

Age:_________

Sex: [M] [F]

PATIENT PARTICIPATION

1. Did the patient consent to participate in the study: [Y] [N]

2. For both the patients that consented into the study AND those that did not consent, please tick which tests were requested by the doctor for the patient:

- [ ] CD4
- [ ] Hb
- [ ] ALT
- [ ] Creatinine

3. For patients who consent into the study please continue to ask the patient the questions on page 2 section 3 below **after taking their blood** for POC testing

3.1. How did it feel after having your finger stick/s?

- ☐ It was fine
- ☐ It hurt
- ☐ I don't know

3.2. Was finger stick worse than having your blood taken from your arm?

- ☐ Yes
- ☐ No
- ☐ It felt the same
- ☐ I don't know

3.3. Please rate the level of pain you experienced for each of the finger sticks, on a **scale of 1 to 4**

(where 1 = no pain; 2 = minimal pain; 3 = mild pain; 4 = severe pain)

CD4 scale_____

Hb scale_____

ALT scale_____

Creatinine scale_____

3.4. Which is most correct to you?

- ☐ I prefer x1 finger stick over x1 needle draw
- ☐ I prefer x2 finger sticks over x1 needle draw
- ☐ I prefer x3 or more finger sticks over x1 needle draw

NURSE COMMENT SECTION

Do you have any additional comments or things you would like to share?

____

____

____
