# Supplementary material for: Human Immunodeficiency Virus (HIV)-Infected Patients Accept Finger Stick Blood Collection for Point-Of-Care CD4 Testing
Source: PLoS One. 2016 Aug 24;11(8):e0161891. doi: 10.1371/journal.pone.0161891 (PMC4996420; doi:10.1371/journal.pone.0161891)
Supplement: S3 File — For preference immediately after finger stick. (PDF) [file pone.0161891.s003.pdf]

Multi-centre evaluation of dedicated and point of care CD4 technologies

Version 1.0 ; 20-NOV-2013

We are interested to hear your experience and remarks after the finger prick. Please fill in this document immediately after the prick, and give it back to the nurses. You can write your final opinion a few days later, on the second document.

Administrative number : ………………………………

Just after the prick (thick one choice)

- ☐ I prefer the usual venipuncture
- ☐ I prefer the finger prick
- ☐ No preference

Why ? (thick one or more choices)

- ☐ Pain/sore
- ☐ Bleeding
- ☐ Difficult to take my sample (by nurse)
- ☐ Risk of infection
- ☐ Visible wound
- ☐ Other : ………………………………………….

  ……………………………………………………
