# Supplementary material for: Human Immunodeficiency Virus (HIV)-Infected Patients Accept Finger Stick Blood Collection for Point-Of-Care CD4 Testing
Source: PLoS One. 2016 Aug 24;11(8):e0161891. doi: 10.1371/journal.pone.0161891 (PMC4996420; doi:10.1371/journal.pone.0161891)
Supplement: S4 File — For preference immediately after finger stick. (PDF) [file pone.0161891.s004.pdf]

Multi-centrum evaluatie van referentie en point of care CD4 technologieën

Version 1.0 ; 20-NOV-2013

We zouden graag weten wat uw mening is over bloedafname door middel van vingerprik. Gelieve dit document in te vullen, onmiddellijk na de prik, en afgeven aan de verplegers. U mag uw finale mening schrijven na een paar dagen op het tweede document.

Administratieve nummer : ……………………………….

Onmiddellijk na de prik (één keuze aanduiden)

- ☐ Ik heb liever de gewone bloedafname
- ☐ Ik heb liever de vingerprik
- ☐ Ik heb geen voorkeur

Waarom ? (één of meerdere keuzes aanduiden)

- ☐ Pijn
- ☐ Bloeding
- ☐ Staal moeilijk te nemen (door de verpleger)
- ☐ Risico van infectie
- ☐ Zichtbare wond
- ☐ Andere : ………………………………………….

  ………………………………………………………
