# Supplementary material for: Human Immunodeficiency Virus (HIV)-Infected Patients Accept Finger Stick Blood Collection for Point-Of-Care CD4 Testing
Source: PLoS One. 2016 Aug 24;11(8):e0161891. doi: 10.1371/journal.pone.0161891 (PMC4996420; doi:10.1371/journal.pone.0161891)
Supplement: S5 File — For preference immediately after finger stick. (PDF) [file pone.0161891.s005.pdf]

Evaluation multicentrique de technologies de référence et Point of care

Version 1.0 ; 20-NOV-2013

Nous aimerions connaître vos impressions et remarques après la piqûre au doigt. Merci de compléter ce document, directement après la piqûre, et le remettre aux infirmiers. Vous pouvez écrire votre avis final après quelques jours, sur le deuxième document.

Numéro administratif : ……………………………….

Juste après la piqûre (cocher une case)

- ☐ Je préfère la ponction veineuse habituelle
- ☐ Je préfère la piqûre au doigt
- ☐ Je n'ai pas de préférence

Pourquoi ? (cocher une ou plusieurs cases)

- ☐ Douleur
- ☐ Saignement
- ☐ Echantillon difficile à prélever (par l'infirmier)
- ☐ Risque d'infection
- ☐ Plaie visible
- ☐ Autre : ………………………………………….

  …………………………………………………….
