# Supplementary material for: Human Immunodeficiency Virus (HIV)-Infected Patients Accept Finger Stick Blood Collection for Point-Of-Care CD4 Testing
Source: PLoS One. 2016 Aug 24;11(8):e0161891. doi: 10.1371/journal.pone.0161891 (PMC4996420; doi:10.1371/journal.pone.0161891)
Supplement: S6 File — For preference two days after finger stick. (PDF) [file pone.0161891.s006.pdf]

Multi-centre evaluation of dedicated and point of care CD4 technologies

Version 1.0 ; 20-NOV-2013

We are interested to hear your experience & remarks after the finger prick. Please fill in this document a few days later, when you don't have any symptoms anymore. You can give this document back at the HIV/SOA reception at your next visit.

Administrative number : ……………………………

A few days after the prick (thick one choice)

- ☐ I prefer the usual venipuncture
- ☐ I prefer the finger prick
- ☐ No preference

Just after the prick, I thought:

- ☐ I prefer the usual venipuncture
- ☐ I prefer the finger prick
- ☐ No preference

Why ? (thick one or more choices)

- ☐ Pain

  Pain duration after venipuncture: ……. hours

  Pain duration after finger prick: ……. hours

- ☐ Bleeding

  Bleeding duration after venipuncture: ……. minutes

  Bleeding duration after finger prick: ……. minutes

- ☐ Wound reopening
- ☐ Infection
- ☐ Visible wound
- ☐ Bulky tape
- ☐ Other : ………………………………………….

  …………………………………………………….

Any other comment or details on your answer (according to your situation etc): ………………………………………

……

……

……
