# Supplementary material for: Human Immunodeficiency Virus (HIV)-Infected Patients Accept Finger Stick Blood Collection for Point-Of-Care CD4 Testing
Source: PLoS One. 2016 Aug 24;11(8):e0161891. doi: 10.1371/journal.pone.0161891 (PMC4996420; doi:10.1371/journal.pone.0161891)
Supplement: S7 File — For preference two days after finger stick. (PDF) [file pone.0161891.s007.pdf]

Multi-centrum evaluatie van referentie en point of care CD4 technologieën

Version 1.0 ; 20-NOV-2013

We zouden graag weten wat uw mening is over bloedafname door middel van vingerprik. Gelieve dit document een paar dagen na de prik in te vullen, als u geen symptomen meer heeft. U mag dit document aan de HIV/SOA receptie afgeven op uw volgende bezoek.

Administratieve nummer : ……………………………

Een paar dagen na de prik (één keuze aanduiden)

- ☐ Ik heb liever de gewone bloedafname
- ☐ Ik heb liever de vingerprik
- ☐ Ik heb geen voorkeur

Onmiddellijk na de prik dacht ik:

- ☐ Ik heb liever de gewone bloedafname
- ☐ Ik heb liever de vingerprik
- ☐ Ik heb geen voorkeur

Waarom ? (één of meerdere keuzes aanduiden)

- ☐ Pijn

  Duur van de pijn na venipuncture: ……. huur

  Duur van de pijn na vingerprik: ……. huur

- ☐ Bloeding

  Duur van de bloeding na venipuncture: ……. minute

  Duur van de bloeding na vingerprik: ……. minute

- ☐ Wond heropend
- ☐ Infectie
- ☐ Zichtbare wond
- ☐ Vervelende tape
- ☐ Andere : ………………………………………….

  ……………………………………………………

Alle andere commentaren of details over uw antwoord (aangezien uw situatie etc):……………………………………

………

………

………
