# Supplementary material for: Human Immunodeficiency Virus (HIV)-Infected Patients Accept Finger Stick Blood Collection for Point-Of-Care CD4 Testing
Source: PLoS One. 2016 Aug 24;11(8):e0161891. doi: 10.1371/journal.pone.0161891 (PMC4996420; doi:10.1371/journal.pone.0161891)
Supplement: S8 File — For preference two days after finger stick. (PDF) [file pone.0161891.s008.pdf]

Evaluation multicentrique de technologies de référence et Point of care

Version 1.0 ; 20-NOV-2013

Nous aimerions connaître vos impressions et remarques après la piqûre au doigt. Merci de compléter ce document après quelques jours, lorsque vous n'avez plus aucun symptômes. Vous pouvez rendre le document à la réception HIV/SOA lors de votre prochaine visite.

Numéro administratif : ……………………………….

<u>Quelques jours après</u> la piqûre (cocher une case)

- ☐ Je préfère la ponction veineuse habituelle
- ☐ Je préfère la piqûre au doigt
- ☐ Je n'ai pas de préférence

<u>Juste après</u>, je pensais :

- ☐ Je préfère la ponction veineuse habituelle
- ☐ Je préfère la piqûre au doigt
- ☐ Je n'ai pas de préférence

Pourquoi ? (cocher une ou plusieurs cases)

- ☐ Douleur

  Durée de la douleur après la ponction veineuse: ……. heures

  Durée de la douleur après la piqûre au doigt: ……. heures
- ☐ Saignement

  Durée du saignement après la ponction veineuse: ……. minutes

  Durée du saignement après la piqûre au doigt: ……. minutes
- ☐ Réouverture de la plaie
- ☐ Infection
- ☐ Plaie visible
- ☐ Sparadrap encombrant
- ☐ Autre : …………………………………………

  ……………………………………………………

Tout autre commentaire ou détail sur vos réponses (par rapport à votre situation etc): ………………………………

………

………

………
